# Conscious sedation for the management of dental anxiety in third molar extraction surgery: a systematic review

**DOI:** 10.1186/s12903-020-01136-0

**Published:** 2020-05-28

**Authors:** Matteo Melini, Andrea Forni, Francesco Cavallin, Matteo Parotto, Gastone Zanette

**Affiliations:** 1 Oral surgery and Implantology - Department of biomedical and neuromotor science (DIBINEM), University of Bologna, Via San Vitale 59, 40125 Bologna, Italy; 2 Sedation and Emergency in Dentistry Human Centered Project (HCP), The holistic treatment of the dental patient – University of Padua, Via Giustiniani 2, 35128 Padova, Italy; 3 Independent statistician, Solagna, Italy; 4 Department of Anesthesia and Pain Management and Interdepartmental Division of Critical Care Medicine, University of Toronto, Toronto, Canada; 5 Department of Neurosciences, Dentistry Section, Chair of Dental Anesthesia, University of Padua, Via Giustiniani 2, 35128 Padova, Italy

**Keywords:** Systematic review, Dental anxiety, Conscious sedation, third molar

## Abstract

**Background:**

Dental anxiety is a condition associated with avoidance of dental treatment and increased medical and surgical risks. This systematic review aims to summarize available evidence on conscious sedation techniques used for the management of Dental anxiety in patients scheduled for third molar extraction surgery, to identify best approaches and knowledge gaps.

**Methods:**

A comprehensive search was conducted including MEDLINE/Pubmed, EMBASE, SCOPUS, clinicaltrials.gov and the Cochrane Database of Systematic Reviews through March 2019. Only randomized controlled trials were included. PRISMA guidelines were followed. Risk of bias was appraised as reported in the Cochrane Handbook for Systematic Reviews of Interventions.

**Results:**

Seventeen RCTs with a total of 1788 patients were included. Some aspects limited the feasibility of a meaningful meta-analysis, thus a narrative synthesis was conducted. Conscious sedation was associated with improvement in Dental anxiety in six studies. One study reported lower cortisol levels with midazolam vs. placebo, while another study found significant variation in perioperative renin levels with remifentanil vs. placebo.

**Conclusions:**

This review found inconclusive and conflicting findings about the role of Conscious sedation in managing Dental anxiety during third molar extraction surgery. Relevant questions remain unanswered due to the lack of consistent, standardized outcome measures. Future research may benefit from addressing these limitations in study design.

## Background

Dental anxiety is a common condition that is associated with avoidance of dental treatment and increased medical and surgical risks [1]. As a physiological response to stressful conditions, it can generate heart rate and blood pressure increase, pallor, excessive sweating, dizziness and lead to a fight or flight response [1]. Dental anxiety is also one of the main factors that impairs dental treatment, thus representing a challenge to professional care [2, 3].

Third molar extraction is a very common oral-surgical intervention and a reproducible surgical stimulus, hence it may represent a valuable scenario for the investigation of conscious sedation techniques [4]. In the last few decades, general anaesthesia has been largely replaced with conscious sedation for third molar extraction. This approach maintains patient comfort while assuring safety and reducing time to discharge [5]. Different medications have been proposed for the management of dental anxiety, such as benzodiazepines, nitrous oxide (N_2_O), opioids, barbiturates, alpha-2 adrenergic receptor agonist, phytotherapics and others [6–10]. Although dental anxiety is a well-known condition that can be managed both with non-pharmacological intervention (e.g., iatrosedation) and pharmacosedative techniques [6, 11], available research on this topic offers a heterogeneous and undefined picture. Anxiety improvement during dental surgery is often included only as secondary outcome in trial protocols, with different outcome measures [10, 12, 13], and endpoints (i.e. before, during and after surgery).

This systematic review aims to summarize available evidence on conscious sedation techniques in the management of dental anxiety in third molar extraction surgery, in order to identify best approaches and gaps in knowledge.

## Methods

### Study design

This is a systematic review of randomized controlled trials (RCTs) evaluating conscious sedation in the management of dental anxiety during third molar extraction surgery. The review was performed following the Preferred Reporting Items for Systematic Reviews and Meta-Analyses (PRISMA) guidelines [14]. This protocol was not registered.

### Search strategy

To identify relevant studies, we systematically searched MEDLINE/PubMed, EMBASE, SCOPUS, Cochrane Central Register of Controlled Trials and Clinicaltrials.gov. The search strategy was carried out without language restrictions from January 1, 1978 until March 31, 2019. In PubMed, the following search strategy was used: “third molar” AND “conscious sedation”. This search strategy was adapted to suit the other electronic sources. The search lists from the electronic sources were merged and the duplicates were removed. Two investigators (MM, AF) independently reviewed the search results and screened both titles and abstracts, to remove the studies outside the scope of the review. We obtained the full texts of all potentially eligible studies, which were further examined to exclude those not fulfilling inclusion criteria. Finally, we hand-searched the reference lists of retrieved articles to identify additional studies of interest. Any inconsistencies were resolved by consensus with a third investigator (MP).

### Criteria for considering studies for this review

Study design: parallel and crossover RCTs.

Population: adult patients (aged 16 or more) undergoing third molar surgery extraction.

Intervention: any type of conscious sedation, including pharmacological intravenous (IV) conscious sedation, pharmacological inhalation conscious sedation (INH), pharmacological orally-administered (OS) conscious sedation, or combination of different techniques of conscious sedation.

Comparator: any type of conscious sedation or no sedation.

Outcome: dental anxiety as reported by patients or assessed using stress hormone alteration; no secondary outcomes were considered.

Time: preoperative, intraoperative and/or postoperative.

Studies not including human subjects were excluded. Studies where patient anxiety was evaluated by an observer were also excluded. No language restrictions were applied.

### Data collection

Two investigators (FC, MP) independently extracted key data from the included articles. For each article, we extracted study features (i.e. study design, year of publication, country, number and age of enrolled patients), type of sedation, and anxiety information (measure of anxiety, timing of assessment, outcomes measures). A third investigator (GZ) checked the extracted data.

### Assessment of risk of bias

Two investigators (FC, MP) independently appraised the risk of bias of the included studies by using the criteria reported in the Cochrane Handbook for Systematic Reviews of Interventions.

Seven specific domains related to risk of bias of RCTs were assessed (random sequence generation; allocation concealment; blinding of participants and personnel; blinding of outcome assessment; incomplete outcome data; selective data reporting; other bias).

Five additional domains related to risk of bias of crossover RCTs were also assessed (whether the cross-over design was suitable; whether there was a carry-over effect; whether only first period data were available; incorrect analysis; comparability of results with those from parallel-group trials).

The risk of bias was categorized as high, low, or unclear as described by the developers [15]; if not available, studies were judged at unclear risk of bias. Any inconsistencies were resolved by consensus with a third investigator (MM).

### Data synthesis

A narrative synthesis of included studies was conducted, because some aspects limited the feasibility of a meaningful meta-analysis. Such aspects included the large number of types of sedation that were evaluated, the heterogeneous measures of anxiety and the heterogeneous timing of assessment of anxiety. Relevant data were extracted from selected studies and variables displayed into tables. The selection process was displayed in a flow-chart.

## Results

### Search results

**Figure 1:**
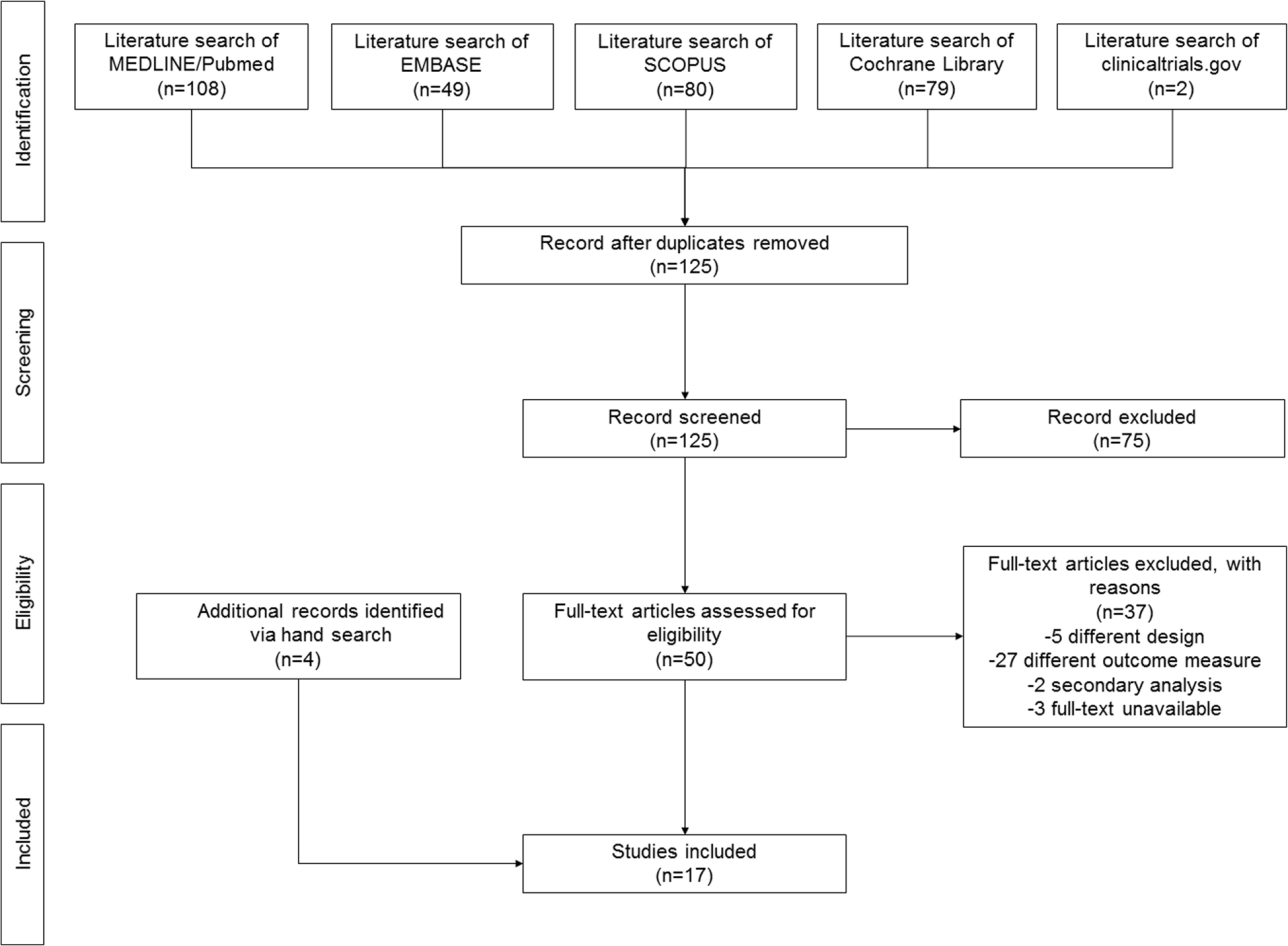
**Fig. 1** PRISMA flow-chart

The search yielded 125 non-duplicated articles. After excluding 75 articles based on title/abstract, 50 articles were retrieved for full text review. Of these, 34 were excluded due to different design (five not RCT), different outcome measures (27 studies not evaluating anxiety or assessing anxiety by external observers), or reporting of secondary analyses (2 studies). Other three studies could not be retrieved (one study from the 80s, one congress abstract without any further publications and one paper published in a journal no longer existing). Four additional articles were identified via hand search, thus a total of 17 RCTs [2, 4, 8–10, 12, 16–26] were included in the qualitative synthesis (Fig. 1).

### Study and patient characteristics

The analysis included seven crossover RCTs and 10 parallel RCTs. Characteristics of included studies are reported in Table 1. The number of enrolled participants ranged from 12 to 997 participants. The drugs that were used for conscious sedation included *Passiflora incarnate*, Midazolam, Dexmedetomidine, N_2_O, Clonidine, Remifentanil, Zaleplon, Triazolam, Chlordemethyldiazepam (CDDZ), Propofol, Fentanyl, and Methohexital. Fourteen studies investigated patient-reported anxiety using different scales: visual analogue scale (VAS), Corah dental anxiety scale (DAS), Interval scale of anxiety report (ISAR), state trait anxiety inventory (STAI); other interval scales. Two studies investigated anxiety by assessing stress hormones alteration. One study investigated anxiety using both patient-reported scale (DAS) and stress hormones alteration (salivary cortisol) (Table 1).

**Table 1:** Characteristics of included studies

#	Study, year	Country	Study design	Enrolled participants, no.	Participant age, years	Types of sedations / Route of administration	Perioperative assessment of anxiety	Measure of anxiety
1	Dantas, 2017 [2]	Brazil	Crossover RCT	40	> 18	-Passiflora incarnate 260 mg (OS)	Postop	DAS
						-Midazolam 15 mg (OS)		
2	Smiley, 2014 [12]	US	Parallel RCT	24	18–32	-Dexmedetomidine 1 μg/kg	Preop, postop	VAS
						+ Midazolam 0.03 mg/kg + Dexmedetomidine infusion 0.07 μg/k/hr. (IV)		
						-Dexmedetomidina 1 μg/kg + placebo + Dexmedetomidina infusion 0.07 μg/k/hr. (IV)		
3	Pereira Santos, 2013 [10]	Brazil	Crossover RCT	32	18–29, with moderate or high anxiety	- N_2_O/O_2_ 50% (INH)	Preop (DAS)	DAS, salivary cortisol
						-Midazolam 7.5 mg (OS)	Preop - postop (salivary cortisol)	
4	Studer, 2012 [16]	Switzerland	Crossover RCT	12	18–40	-Midazolam 7.5 mg (OS)	Preop - Postop	VAS
						-Clonidine 150 μg (OS)		
5	Esen, 2005 [17]	Turkey	Crossover RCT	20	18–26	-Midazolam 0.05 mg/kg + PCI Remifentanil (20 μg *initial bolus* + 3 μg ・kg − ^1^ ・h − ^1^*continuous infusion +* 15 μg *PCI, lockout 5 min, max 500 μg/h)* (IV)	Preop, intraop, postop	Aldosterone, ACTH, renin
						-Midazolam 0.05 mg/kg + placebo (IV)		
6	Fong, 2005 [18]	China	Parallel RCT	40	> 18	-Remifentanil 15–20 μg/ml *PCI 1 ml bolus infused over 30 s, no further lockout* (IV)	Postop	VAS
						-Placebo (IV)		
7	Ganzberg, 2005 [9]	US	Crossover RCT	14	18–40	-Zaleplon 10 mg (OS)	Preop, postop	VAS
						-Triazolam 0.5 mg (OS)		
8	Jerjes, 2005 [19]	UK	Parallel RCT	46	> 18	-Midazolam 7.5 mg (OS)	Preop, intraop, postop	Salivary cortisol
						-Placebo (OS)		
9	Manani, 2004 [20]	Italy	Parallel RCT	50	N/A	-CDDZ 1 mg + midalozam 1 mg (OS+IV)	Postop	VAS, ISAR
						-CDDZ 1 mg + midalozam 2 mg (OS+IV)		
10	Leitch, 2004 [4]	UK	Parallel RCT	110	17–49	-Midazolam 2 mg + 1 mg *per minute until patient-reported readiness* (IV)	Preop, Intraop	VAS
						-Propofol 1% PMS – *initial plasma concentration 1.5 μg/ml, then 1.0 μg/ml with patient controlled increments of 0.2 μg/ml lockout 2 min* (IV)		
11	Dionne, 2001 [8]	US	Parallel RCT	997	N/A	-Midazolam *until conscious sedation (mean dose 8.6 mg)* (IV)	Intraop, postop	Scale 2–42
						-Midazolam *until conscious sedation* + additional midazolam *during surgery (mean total dose 12.2 mg)* (IV)		
						-Fentanyl (1.4 μg/kg) + Midazolam *until conscious sedation (mean dose 5.5 mg)* (IV)		
						-Fentanyl (1.4 μg/kg) + Midazolam *until conscious sedation (mean dose 5.8 mg)* + additional Methohexital during surgery (mean dose 61.0 mg) (IV)		
						-Placebo (IV)		
12	Bell, 2000 [21]	UK	Parallel RCT	60	19–55	-Midazolam titrated in increments of 1 mg or 2 mg every 1 min (IV) + local anesthesia	Postop	DAS
						-Local anesthesia		
13	Milgrom, 1994 [22]	US	Parallel RCT	207	> 18	-Midazolam *titrated 1 mg per minute* (IV)	Intraop, postop	Scale 0–42
						-Midazolam *titrated 1 mg per minute pre-surgery* + Midazolam *titrated 1 mg per minute during surgery up to 10 mg* (IV)		
						-Fentanyl 0.014 mg/kg + Midazolam *titrated 1 mg per minute* (IV)		
						-Fentanyl 0.014 mg/kg + Midazolam *titrated 1 mg per minute* + methohexital *10 mg bolus* + Methohexital *follow up dose during surgery up to 200 mg* (IV)		
						-Placebo (IV)		
14	Stopperich, 1993 [23]	US	Parallel RCT	22	18–35	-Triazolam 0.25 mg (OS)	Preop	VAS, ISAR
						-Placebo (OS)		
15	Luyk, 1992 [24]	New Zealand	Crossover RCT	41	18–40	-Midazolam 1 mg/min (IV)	Postop	VAS
						-Placebo (IV)		
16	Luyk, 1991 [25]	New Zealand	Crossover RCT	33	18–32	-Midazolam 7.5 mg (OS) + midazolam (IV)	Intraop	VAS
						-Placebo (OS) + midazolam (IV)		
17	O’Boyle, 1987 [26]	Ireland	Parallel RCT	40	N/A	-Midazolam 15 mg (OS) + placebo (IV)	Postop	VAS, STAI
						-Diazepam 10 mg (IV) + placebo (OS)		

*RCT* Randomized controlled trial, *N/A* Not available, *Intraop* Intraoperative, *Preop* Preoperative, *Postop* Postoperative, *VAS* Visual analogue scale, *DAS* Corah dental anxiety scale, *ISAR* Interval scale of anxiety report, *STAI* State trait anxiety inventory, *ACTH* Adrenocorticotropic hormone

### Risk of bias in included studies

The risk of bias is reported in Table 2. The risk of selection bias was low in five studies (adequate random sequence generation) and unclear in the others with regards to random sequence generation, while it was unclear in all studies with regards to allocation concealment (which was never specified). The risk of performance bias was low in 12 studies (blinded participants and personnel) and unclear in five. The risk of detection bias was low in four studies (blinded outcome assessor) and unclear in 13. Four studies [10, 12, 16, 25] were at high risk of attrition bias. In Smiley et al. [12], one patient in the dexmedetomidine-only arm withdrew from the study citing inadequate sedation. In Pereira Santos et al. [10], two patients did not return for the second surgery and two were excluded due to signs and symptoms of over-sedation. In Studer et al. [16], two patients withdrew from the study citing excessive anxiety. In Luyk et al. [25], two patients did not return for the second surgery. The risk of attrition bias was low in 11 studies (no dropouts or missing data) and unclear in two (no information on dropouts or missing data). The risk of reporting bias was low in all studies. Four studies [2, 10, 16, 24] were at high risk of other bias due to incorrect analysis of data from crossover designs (i.e. paired tests were performed but the period effect could not be removed because of different sequence sizes).

**Table 2:** Risk of bias summary

#	Study, year	Random sequence generation (selection bias)	Allocation concealment (selection bias)	Blinding of participants and personnel (performance bias)	Blinding of outcome assessment (detection bias)	Incomplete outcome data addressed (attrition bias)	Selective outcome reporting (reporting bias)	Other bias
1	Dantas, 2017 [2]	Unclear	Unclear	Low	Low	Low	Low	High
2	Smiley, 2014 [12]	Unclear	Unclear	Low	Low	High	Low	Low
3	Pereira Santos, 2013 [10]	Unclear	Unclear	Unclear	Unclear	High	Low	High
4	Studer, 2012 [16]	Low	Unclear	Low	Unclear	High	Low	High
5	Esen, 2005 [17]	Unclear	Unclear	Low	Unclear	Low	Low	Low
6	Fong, 2005 [18]	Unclear	Unclear	Low	Low	Low	Low	Low
7	Ganzberg, 2005 [9]	Unclear	Unclear	Low	Unclear	Unclear	Low	Low
8	Jerjes, 2005 [19]	Unclear	Unclear	Low	Low	Low	Low	Low
9	Manani, 2004 [20]	Unclear	Unclear	Unclear	Unclear	Low	Low	Low
10	Leitch, 2004 [4]	Low	Unclear	Low	Unclear	Low	Low	Low
11	Dionne, 2001 [8]	Unclear	Unclear	Unclear	Unclear	Low	Low	Low
12	Bell, 2000 [21]	Low	Unclear	Unclear	Unclear	Low	Low	Low
13	Milgrom, 1994 [22]	Low	Unclear	Low	Unclear	Low	Low	Low
14	Stopperich, 1993 [23]	Unclear	Unclear	Low	Unclear	Low	Low	Low
15	Luyk, 1992 [24]	Unclear	Unclear	Low	Unclear	Unclear	Low	High
16	Luyk, 1991 [25]	Unclear	Unclear	Unclear	Unclear	High	Low	Low
17	O’Boyle, 1987 [26]	Low	Unclear	Low	Unclear	Low	Low	Low

### Narrative synthesis on patient-reported anxiety

All but two studies [17, 19] investigated patient-reported anxiety (Table 3).

**Table 3:** Patient-reported anxiety (narrative synthesis)

#	Study, year	VAS	DAS	Other scale
1	Dantas, 2017 [2]	NA	Unclear findings on anxiety: the data suggested a period effect that was not tested and was not removed by the design due to different sequence size	NA
2	Smiley, 2014 [12]	The study was underpowered (suggesting lower anxiety with Midazolam vs. placebo)	NA	NA
3	Pereira Santos, 2013 [10]	NA	Unclear findings on anxiety: the data suggested a period effect that was not tested and was not removed by the design due to different sequence size	NA
4	Studer, 2012 [16]	No statistically significant difference between Midazolam vs. Clonidine	NA	NA
6	Fong, 2005 [18]	No statistically significant difference in the increase in anxiety score during operation between remifentanil vs. placebo	NA	NA
7	Ganzberg, 2005 [9]	The study was underpowered (suggesting lower anxiety with Zaleplon vs. Triazolam)	NA	NA
9	Manani, 2004 [20]	Lower anxiety with Midazolam 2 mg vs. 1 mg	Lower anxiety with Midazolam 2 mg vs. 1 mg	NA
10	Leitch, 2004 [4]	Lower anxiety with Propofol vs. Midazolam	NA	NA
11	Dionne, 2001 [8]	NA	NA	Lower anxiety with any intervention group vs. placebo; lower intraoperative anxiety with Fentanyl + Midazolam vs. Midazolam; lowest anxiety with Fentanyl + Midazolam + Methohexital vs. any other group
12	Bell, 2000 [21]	NA	Lower anxiety with Midazolam vs. none	NA
13	Milgrom, 1994 [22]	NA	NA	Lower anxiety with Fentanyl + Midazolam or with Fentanyl + Midazolam+ Methohexital + Methohexital
14	Stopperich, 1993 [23]	Lower anxiety with triazolam vs. placebo	Lower anxiety with triazolam vs. placebo	NA
15	Luyk, 1992 [24]	No statistically significance different between midazolam vs. placebo	NA	NA
16	Luyk, 1991 [25]	Lower anxiety with midazolam vs. placebo	NA	NA
17	O’Boyle, 1987 [26]	Lower anxiety with midazolam vs. diazepam	NA	No statistically significance different between midazolam vs. diazepam

Six studies found some statistically significant differences between arms. One study reported lower anxiety with triazolam vs. placebo [23], while one study reported lower anxiety with midazolam vs. no intervention [21].

One study reported lower anxiety with midazolam 2 mg vs. 1 mg [20], and one study reported lower anxiety with propofol vs. midazolam [4]. One study reported lower anxiety with any intervention group vs. placebo, lower intraoperative anxiety with fentanyl + midazolam vs. midazolam, while subjects receiving fentanyl + midazolam + methohexital reported the lowest anxiety [8]. One study reported lower anxiety with fentanyl + midazolam or with fentanyl + midazolam+ methohexital with respect to placebo or midazolam [22].

In another study, lower anxiety with midazolam vs. diazepam was shown by using VAS but not with STAI scale [26].

Four studies reported unclear findings on anxiety assessment / evaluation or were underpowered to draw definitive conclusions. One study comparing *Passiflora incarnate* vs. Midazolam [2] and another comparing midazolam vs. N_2_O/O_2_ [10] reported unclear findings on anxiety, since the data suggested a period effect that was not tested and was not removed by the design due to different sequence size. Other two studies [9, 12] suggested lower anxiety with midazolam vs. placebo, and with zaleplon vs. triazolam, but were underpowered to achieve statistical significance.

Two studies [16, 18] did not find any statistically significant difference between midazolam vs. clonidine, and between remifentanil vs. placebo.

Luyk et al. found no statistically significant difference in postoperative anxiety between midazolam vs. placebo in 1992 [24], while they reported lower intraoperative anxiety with midazolam administered orally and intravenously vs. oral placebo + intravenous midazolam (attrition bias) in 1991 [25].

### Narrative synthesis on anxiety by assessing stress hormones alteration

Three studies [10, 17, 19] investigated anxiety by assessing stress hormone alteration (Table 4). One study [19] reported lower cortisol levels with midazolam vs. placebo, while unclear findings were reported by another study comparing midazolam vs. N_2_0/O_2_ [10]. One study [17] found significant variation in perioperative renin levels with remifentanil vs. placebo, while aldosterone and ACTH levels were not statistically different.

**Table 4:** Stress hormone alteration (narrative synthesis)

#	Study, year	Cortisol	Aldosterone	ACTH	Renin
3	Pereira Santos, 2013 [10]	Unclear findings on anxiety: only first period data were available but were not compared	NA	NA	NA
5	Esen, 2005 [17]	NA	No statistically significant difference between remifentanil vs. placebo	No statistically significant difference between remifentanil vs. placebo	Higher preoperative to intraoperative increase and intraoperative to postoperative decrease in remifentanil vs. placebo
8	Jerjes, 2005 [19]	Lower levels with midazolam vs. placebo at preoperative, intraoperative and postoperative	NA	NA	NA

*ACTH* Adrenocorticotropic hormone, *NA* Not available

## Discussion

This review found inconclusive and conflicting findings about the role of conscious sedation in the management of dental anxiety during third molar extraction surgery. Summary of the findings was limited by the lack of a of consistent, standardized outcome measures, thus preventing from drawing definitive conclusions.

Dental anxiety is a common aspect of oral-surgical procedures, and patients may benefit from the use of conscious sedation [10]. The advantages of conscious sedation may include lower patient anxiety [8, 19, 21, 23, 25], reduced post-surgical pain [27] increased patient and surgeon satisfaction [28], and inhibition of gag reflex [29]. In addition, it can be safely delivered in a non-operating room environment [30] and to patients with challenging behaviors as an alternative to general anesthesia [5]. Third molar extraction is a very common oral-surgical procedure that is very frequently associated with anxiety, thus presenting a large opportunity for the application of conscious sedation [4]. Although many studies have investigated this topic, the best approach remains to be established. Available literature shows large heterogeneity in selection of sedation drugs (or drugs combination), route of administration, anxiety evaluation scales and timing of assessment.

Patient-reported anxiety was the preferred outcome measure (despite being assessed using different scales), with six studies providing some indications from the comparison of different drugs [4, 8, 20–23] and nine studies reporting unclear or inconclusive findings [2, 9, 10, 12, 16, 18, 24–26]. Lower cortisol levels were associated with midazolam [19] and remifentanil [17] when compared to placebo, while unclear findings were reported in the comparison of midazolam with N_2_0/O_2_ [10].

The findings of this review should be interpreted within its limitations. First, the quality of included studies was unclear regarding some domains and some studies were at high risk of bias due to incorrect analysis of data from crossover design. Second, heterogeneity in comparisons, assessment and outcome measures prevented from pooling the findings of the included studies. Third, the focus on third molar extraction does not allow to generalize our considerations to other oral-surgical procedures.

Regarding the applicability of evidence, the use of pharmacological conscious sedation did not consistently show any advantages in the management of dental anxiety in third molar extraction surgery. There are still relevant questions that remain unanswered, including i) what the preferred drugs (and route of administration) should be, ii) what set of outcome measures should be included, and iii) when the outcome measures should be assessed. Of note, another open issue involves the investigation of sedation procedures separately from psychological and behavioral evaluations. A previous study showed a long-term reduction of dental fear in children thanks to behavioral and cognitive-behavioral interventions, while conscious sedation was less effective [31]. Furthermore, there was no strong evidence of anxiolytic effects of specific drugs such as opioids, which can lead to difficult social management if overprescribed. The justification for routine use of such drugs remains unclear, given the availability of less hazardous alternatives [32]*.*

Although this review cannot draw definitive conclusions, the findings highlight some implications for future research on the topic. The definition of a minimum degree of standardization in study design can provide several benefits in terms of comparability and generalizability of the findings. Of note, crossover design can be a valuable approach but requires appropriate implementation (i.e. randomization of sequences and adequate analysis of different-size sequences) [15]. A critical choice of investigated drugs could be advisable, since considerable information on safety and anxiolytic effectiveness of different molecules is available in literature. In our opinion, priority should be given to drugs that showed better anxiolytic effect and lower risk of adverse effects, easiness of use and higher patient satisfaction. An ordered sequence of possible comparisons may be defined for future investigations, thus leading to increased evidence of a low-level comparison before moving to the next-level comparison. In addition, a placebo-controlled arm should also be preferred to a no-intervention arm. A list of clinically relevant and validated outcome measures may be agreed upon a priori, in order to allow comparability and generalizability of the findings. Dental anxiety being a subjective state, outcome measures may include patient-reported anxiety (i.e. VAS, DAS) and objective parameters (i.e. stress hormones alteration), while external observer’s assessment should be avoided (such studies were excluded from the review). The timing of anxiety evaluation should include baseline (i.e. few days before surgery, during the pre-operative visit), before-sedation and after-sedation (but before local anesthetic injection) because postoperative assessments are associated with a spontaneous anxiety reduction [33]. In addition, intra-operative data regarding not only standard physiological parameters (such as heart rate, blood pressure, peripheral capillary oxygen saturation) but also less used ones (such as the relative parasympathetic tone assessed by Analgesia/Nociception Index (ANI) should be collected to evaluate the overall patient’s perception of the surgical experience [34, 35].

## Conclusions

This review found inconclusive and conflicting reports about the role of conscious sedation in managing dental anxiety in third molar extraction surgery. Relevant questions remain unanswered due to the lack of consistent, standardized outcome measures. Future research may benefit from a systematic standardization in study design.

## Data Availability

The datasets generated during and/or analysed during the current study are available from the corresponding author on reasonable request.

## Abbreviations

CDDZ: Chlordemethyldiazepam
DAS: Corah dental anxiety scale
INH: Inhalation conscious sedation
ISAR: Interval scale of anxiety report
RCT: Randomized clinical trials
STAI: State trait anxiety inventory
VAS: Visual analogue scale
